# A Review of Salivary Diagnostics and Its Potential Implication in Detection of Covid-19

**DOI:** 10.7759/cureus.7708

**Published:** 2020-04-17

**Authors:** Tatikonda Sri Santosh, Reshu Parmar, Hanish Anand, Konkati Srikanth, Madham Saritha

**Affiliations:** 1 Orthodontics and Dentofacial Orthopaedics, Malla Reddy Institute of Dental Sciences, Hyderabad, IND; 2 Orthodontics and Dentofacial Orthopaedics, Pandit Deendayal Upadhyay Dental College, Solapur, IND; 3 Orthodontics and Dentofacial Orthopaedics, Panineeya Mahavidyalaya Institute of Dental Sciences and Research, Hyderabad, IND; 4 Orthodontics and Dentofacial Orthopaedics, Family Dental Care, Hyderabad, IND

**Keywords:** salivary diagnostics, safe, non invasive, cost effective, novel corona virus

## Abstract

Saliva is an exocrine secretion produced from the salivary glands and has numerous functions, such as cleansing and protection of the oral cavity, antimicrobial effects and aids in digestion. Due to the speedy development in the field of salivaomics, saliva is now well accepted as a pool of biological markers that vary from changes in biochemicals, nucleic acids and proteins to the microflora. Saliva has an immense potential as a diagnostic fluid and offers an edge over other biological fluids as its collection method does not require invasive procedure, economical and is useful for monitoring systemic health. Development of sensitive and precise salivary diagnostic tools and the formulation of defined guidelines following meticulous testing will allow salivary diagnostics to be utilised as chair side tests for various oral and systemic diseases in the near future.

The coronavirus disease (Covid-19) pandemic is the biggest challenge and global health crisis for the world since World War Two. Rapid and accurate diagnosis of Covid-19 is crucial in controlling the outbreak in the community and in hospitals. Nasopharyngeal and oropharyngeal swabs are the recommended specimen types for Covid-19 diagnostic testing. The collection of these specimen types requires close contact between healthcare workers and patients and poses a risk of transmission of the virus, causes discomfort and may cause bleeding, especially in patients with condition such as thrombocytopenia. Hence, nasopharyngeal or oropharyngeal swabs are not desirable for sequential monitoring of viral load. Saliva specimens can be obtained easily as the patient is asked to spit into a sterile bottle. The collection of saliva is non-invasive and greatly minimizes the exposure of healthcare workers to Covid-19. Saliva has a high consistency rate of greater than 90% with nasopharyngeal specimens in the detection of respiratory viruses, including coronaviruses.

Saliva has also been used in screening respiratory viruses among hospitalized patients without pyrexia or respiratory symptoms. SARS-CoV can be detected in saliva at high titers. Salivary diagnostics is a dynamic field that is being incorporated as part of disease diagnosis, clinical monitoring of systemic health and to make significant clinical decisions for patient care. More research is required to analyze the potential diagnostic of Covid-19 in saliva to develop rapid chair side tests for the detection of Covid-19 and it is also pivotal to improve and develop successful strategies for prevention, especially for dentists and healthcare professionals who are involved in performing aerosol-generating procedures.

## Introduction and background

Saliva is a hypotonic fluid in nature. The major salivary glands such as the parotid glands, submandibular glands and sublingual glands secrete approximately 90% of saliva. The salivary glands have high permeability and are surrounded by abundant capillaries, blood and acini, which can exchange molecules. Hence, biomarkers in the blood circulation can infiltrate acini and ultimately secreted into the saliva [1]. Every day, 600 ml of serous and mucinous saliva is secreted from the human salivary glands which contains minerals, electrolytes, buffers, enzymes and enzyme inhibitors, growth factors and cytokines, immunoglobulins (e.g., secretory immunoglobulin A [IgA]), mucins and other glycoproteins [2]. Saliva has been studied thoroughly as a potential diagnostic tool and it is expected to become a substitute for other biological fluids such as serum or urine in disease diagnosis.

Advantages of salivary testing for diagnosis are as follows [3-6]:

Non-invasive, economical. Safer to administer than serum sampling (no needles). Real-time diagnostic values. No need for trained medical staff. Multiple samples can be obtained easily. Collection and screening can be done at home. Minimizes the risks of cross-contamination. More economical sampling, shipping and storage compared to serum. Requires lesser manipulation during diagnostic procedures compared to serum. Commercial availability of screening assays. Saliva does not clot and can be manipulated more easily than blood.

Emerging latest technologies have disclosed large numbers of medically important salivary biomarkers for various disease conditions including cancer, autoimmune, viral, bacterial, cardiovascular, and metabolic diseases [7]. Covid-19 is associated with human to human transmission and was recently detected in the saliva of infected patients. Saliva can have a significant role in the human-to-human transmission, salivary diagnostics may provide an easy and cost-effective point-of-care platform for quick and early diagnosis of Covid-19 [8].

## Review

Human diseases such as cancer, cardiovascular, metabolic, infectious and neurological diseases, have global impact. Diagnosis of these conditions is very demanding and needs supplementing clinical analysis with laboratory testing [4]. Saliva is a complex fluid comprising of proteins, enzymes, hormones, antibodies, cytokines and antimicrobial constituents [7]. The process of entry of these constituents from the blood into the saliva is by transcellular, passive intracellular diffusion and active transport, or paracellular routes by extracellular ultrafiltration within the salivary glands or through the gingival crevice [9,10]. Saliva is colourless, odourless and has a relative density of 1.004-1.009 and a pH of 6.6-7.1. Salivary fluid is an exocrine secretion comprising of approximately 99% water, containing a variety of electrolytes and proteins, represented by enzymes, immunoglobulins and other antimicrobial factors which are of importance to oral health. Saliva protects the teeth and the oro‑esophageal mucosa through a number of mechanisms. Besides maintaining the integrity of these tissues, saliva also has multiple functions in relation to digestion in the upper gastrointestinal tract. This action occurs due to the presence of the digestive enzyme α-amylase (ptyalin) which divides the starch into maltose, maltotriose, and dextrins. Lubrication of oral surfaces, tooth mineralization, buffering, and antimicrobial activity are other beneficial effects of saliva. It is believed that changes in saliva are indicative of wellness of our health.

Saliva in the diagnosis of diseases

In view of the rapid development made in salivary studies, the concept of salivaomics was proposed. Salivaomics involves genomics, transcriptomics, proteomics, metabonomics and microRNA (miRNA) analysis. Wong was responsible for setting up a professional salivaomics knowledge base (SKB) that can systematically manage the data of research related to salivaomics [11]. The limitations concerning the usage of saliva for diagnosis due to its low concentration of analytes compared to blood have been overcome with the development of precise molecular methods and nanotechnology [12].

Caries

The prevalence of dental caries is directly proportional to the microbial load of Streptococcus mutans and Lactobacillus in the saliva. Samaranayake used paraffin wax to stimulate the production of the salivary samples which were incubated in a selective growth media for up to 24 hours [13]. Saliva from populations with high caries activity contained >1 × 10^6^ mL^-1^ of S. mutans and/or 1 × 10^5^ mL^-1^ of Lactobacillus whereas populations with low caries activity harboured <1 × 10^5^ mL^-1^ of S. mutans and/or 1 × 10^4^ mL^-1^ of Lactobacillus.

The presence of both the pathogens in high titres using a commercially available test (CRT® bacteria, Ivoclar-Vivadent Inc., Amherst, MA, USA) has shown a positive correlation with the presence of caries in children and adults [14]. The prognostication of caries susceptibility was assessed with an experimental assay using biomarkers (genetically determined oligosaccharides profiles present on salivary glycoproteins). Saliva also protects against caries since it contains antibacterial agents, can mechanically clear the pathogens and has a buffering capacity to decrease the acid concentration on tooth surfaces. Hence, alterations in quantity and composition of saliva can also provide potential tools to detect and monitor caries.

Periodontal Diseases

Periodontal disease is a chronic inflammatory process of the periodontium in response to bacterial plaque deposited on the teeth. Bacterial infections forming biofilms, induce gingivitis, destroy the alveolar bone and periodontal ligament, cause apical migration of the epithelial attachment resulting in the formation of periodontal pockets, and induce irreversible loss and exfoliation of the teeth. Porphyromonas gingivalis is a ‘red complex’ bacteria that is closely associated with periodontitis. P. gingivalis saliva kit which is based on an enzyme-linked immunosorbent assay have been developed by researchers to specifically detect this bacterium in saliva. The kit can detect both laboratory and clinical isolate strains of P. gingivalis at concentrations of 5 × 10^4 ^to 5 × 10^5^ CFU·mL^-1^ and yields results quickly within 90 seconds. When compared with real-time polymerase chain reaction the P. gingivalis kit is faster in detection and has a sensitivity and a specificity of 92% and 96%, respectively [15]. Isolation of bacterial species from oral specimens using classical in vitro methods is only possible for cultivable species such as Porphyromonas gingivalis, Treponema denticola and Tannerella forsythia present as a complex biofilm in destructive periodontitis.

Several salivary biomarkers have been studied for the diagnosis and prognostication of periodontal diseases. These include inflammatory mediators, enzymes, epithelial keratins, immunoglobulins, salivary ions and hormones. Gingival crevicular fluid (GCF) has been used to detect these potential biomarkers. Specifically, the presence of matrix metalloproteinase-8 (MMP-8, an enzyme responsible for tissue destruction) in GCF has been positively linked with periodontal disease progression [16]. In 2010, an immunochromatographic chair-side dip-stick test became commercially available to determine the presence or absence of MMP-8 in the GCF [17]. It has been reported that there is a positive association between salivary soluble toll-like receptor-2 and interleukin-4 and periodontal disease [18]. Levels of aspartate aminotransferase (AST) and alkaline phosphatase (ALP) are elevated in periodontal diseases. Salivary AST can be used as a marker for monitoring periodontal disease.

According to researchers, variation in more than 70 genes can be attributed to periodontal diseases [19]. Hence, salivary genomics represent another fascinating platform for the diagnosis of periodontitis. After analysing DNA obtained from patients' saliva, it was recently validated that genetic mutations of the IL-6 gene (Cytokine involved in periodontal tissue destruction) are a significant risk factor for chronic periodontitis in Caucasians [20]. Additional research to isolate genetic, microbial and host-derived risk factors will elucidate more on potential biomarkers for periodontal diseases.

Oral Cancer and Systemic Malignancies

Almost in all types of cancer the prerequisite to good prognosis is the early diagnosis. Salivary constituents such as proteins, mRNA, and DNA have been used in the diagnosis of Oral Squamous Cell Carcinoma (OSCC). Somatic mutations of tumour-specific DNA are responsible for the onset and development of malignancy, which can be detected in the saliva or other body fluids and can also be used as biomarkers to diagnose oral or other tumours. In saliva, tumour-specific DNA was positive in 100% of patients with oral tumours whereas, only 47%-70% of patients with other tumours carry tumour-specific DNA in the saliva. In contrast, tumour-specific DNA was found in 80% of plasma samples from patients with oral tumours and in 86%-100% of patients with tumours in other sites which validates the use of saliva as a potential tool to diagnose oral cancers [21].

OSCC is the most common form of cancer affecting oral cavity. Researchers have found that salivary levels of specific proteins such as, CD44 (a cell surface glycoprotein involved in cell-to-cell interaction), Cyfra 21-1 (a fragment of cytokeratin 19), tissue polypeptide antigen (TPS), and cancer antigen 125 (CA-125) are increased in patients with OSCC and have been suggested as oral cancer biomarkers [22]. The use of 7 OSCC-associated saliva RNAs (transcriptomes) has shown a prediction accuracy rate of 81%, indicating their potential for oral carcinoma detection [23].

Mutations of the tumour suppressor p53 were first described for salivary gland adenomas in 1992 and later in a pilot study of saliva from breast cancer subjects [24]. Few studies suggest elevated levels of the cancer antigen, CA15-3 and the oncogene c-erB2, in woman with breast cancer as compared to controls. Chen et al. identified the tumour marker C125 in saliva of subjects with malignant ovarian tumours [25].

Lung Cancer

Mutations identified in the EGF receptor (EGFR) are the tumour-specific biomarkers for non-small cell lung carcinoma (NSCLC). A unique core technology known as electric field-induced release and measurement depends on a multiplexible electrochemical sensor that can detect EGFR mutations in bodily fluids was shown to be effective for the detection of EGFR mutations in the saliva of patients with NSCLC [26]. This implies that proteomic biomarkers could be the key for the early diagnosis and prognosis of lung cancer.

Prostate Cancer

Prostate specific antigen (PSA) is a useful marker for detecting prostate adenocarcinoma (PA), monitoring the treatment or assessing its recurrence. Salivary PSA levels correlate with serum PSA levels in patients with prostate adenocarcinoma. miR-21 and miR-141 are two tumour biomarkers whose levels are significantly elevated in patients with early-stage and advanced-stage prostate cancer, respectively. The expression of both these tumour markers in the saliva can be detected by nano-graphene oxide which makes it a non-invasive approach to diagnose early-stage prostate cancer [27].

Autoimmune Disorders

Sjogren's syndrome (SS) is a chronic autoimmune disorder characterized by reduced secretion of the salivary glands and lacrimal glands and associated endocrine disturbance. Salivary secretion from individuals affected with Sjogren's syndrome demonstrates elevated levels of antibodies and cytokines such as IgA, IgG, prostaglandin-E2, and interleukin-6 along with reduction in oral phosphate levels and xerostomia due to reduced salivary flow, which may result in causing infections, caries, difficulty in swallowing and oral pain [28]. Salivary protein analysis indicates an increased level of lactoferrin, beta-2 microglobulin, lysozyme C, and cystatin C, and decreased levels of salivary amylase and carbonic anhydrase [29].

Cystic fibrosis (CF) is an inherited disorder which is caused due to a mutation in the cystic fibrosis transmembrane conductance regulator (CFTR) gene. The CFTR protein is expressed in the epithelial cells of the parotid gland inflicting its involvement. CF is most frequently seen in Caucasians, usually results in early death from respiratory complications. Saliva of CF patients has elevated levels of calcium and phosphate, which may explain higher incidence of calculus observed in such individuals. These patients also possess higher salivary levels of Cathepsin-D, chloride, potassium and sodium ions with a lower salivary volume and pH compared to healthy individuals. In addition, saliva samples in younger CF patients have been found to have higher levels of proteins, antioxidants and uric acid compared to their normal counterparts [29].

Diabetes

Diabetes is a metabolic disease caused by lack of insulin secretion, insulin action or insulin resistance, which impairs the body’s ability to process blood glucose. A positive association found between α-2-macroglobulin and HbA1c, demonstrated that levels of α-2-macroglobulin in the saliva could reflect the glycaemic control in patients with type 2 diabetes mellitus [30]. Type 1 diabetic hyperglycemia requires measuring exhaled methyl nitrate [31]. Investigations indicated an association between blood glucose levels and exhaled methyl nitrate, presumptively because of interaction of superoxide dismutase with nitric oxide as a byproduct of elevated oxidative reactions. Use of gingival crevicular blood as a measure of blood glucose was proposed by Strauss et al. In a study of 54 subjects, blood collected from GCF was compared to blood obtained with a finger-stick; results showed good correlation between samples collected from the two sites [32].

Cardiovascular Disease

Cardiovascular disease (CVD) is the group of disorders of heart and blood vessels and includes diseases such as atherosclerosis, myocardial infarction and coronary heart disease. The research done by Kosaka et al. revealed significantly elevated levels of salivary inflammatory cytokines including IL-1β, IL-6, TNF-α and prostaglandin E2 in both atherosclerosis and periodontal diseases. These cytokines can be potential biomarkers for the diagnosis of periodontal disease and atherosclerosis [33]. Salivary markers of cardiovascular diseases include C-reactive protein (CRP), myoglobin (MYO), creatinine kinase myocardial band (CKMB), cardiac troponins (cTn), and myeloperoxidase, when used in combination with an ECG, shows a positive association with myocardial infarct patients as compared to healthy controls [29].

CRP protein is an inflammatory mediator that is released in response to acute injury or infection. It can lead to atherogenesis. Significantly, salivary CRP levels were found to correlate with plasma CRP levels obtained from blood samples of a population at risk for cardiovascular complications [34]. Cardiac troponin (CTn), a biomarker for the diagnosis of acute myocardial infarction can be detected in saliva which is released in response to cardiac cell necrosis [35]. Salivary CTn levels were shown to be a sensitive diagnostic tool in patients suffering from acute myocardial infarction [36]. More clinical studies are needed to validate salivary biomarkers for cardiovascular diseases.

Viral Infections

Diagnosis of viral infections presently depends on salivary biomarkers, such as viral DNA and RNA, antigens and antibodies. There are saliva-based antibody tests to detect several viruses. The Raffaele Scientific Institute in Milan used a salivary test named OraQuick® hepatitis C virus rapid antibody test, to detect the hepatitis C virus [37]. The dengue virus (DENV) RNA and non-structural protein 1 antigens are detectable from saliva, which might provide an effective way to diagnose dengue. Salivary levels of anti-dengue IgM and IgG revealed sensitivity of 92% and specificity of 100% in the diagnosis of infection. Nefzi et al. found that saliva seems to be more sensitive than the blood in the detection of HHV-6 or human cytomegalovirus [38]. Salivary tests using polymerase chain reaction for the detection of human papilloma virus have been established.

COVID-19

Coronavirus disease (COVID-19) is a kind of viral pneumonia with its epicentre in Wuhan, China. It is caused by severe acute respiratory syndrome coronavirus 2 (SARS-CoV-2). The unfolding of SARS-CoV-2 has been recognised as the third introduction of a highly pathogenic coronavirus into the human population after the severe acute respiratory syndrome coronavirus (SARS-CoV) and the Middle East respiratory syndrome coronavirus (MERS-CoV) in the twenty-first century [39]. Similar to SARS-CoV, Covid-19 can be efficiently transmitted between humans via droplets and fomites during close unprotected contact between infector and infectee.

Coronaviruses are enveloped viruses with a positive sense single-stranded RNA genome (26-32 kb) [40]. Four coronavirus genera (α, β, γ, δ) have been identified so far, with human coronaviruses (HCoVs) detected in the α coronavirus (HCoV-229E and NL63) and β coronavirus (MERS-CoV, SARS-CoV, HCoV-OC43 and HCoV-HKU1) genera [41]. Coronavirus S protein has been reported as a significant determinant of virus entry into host cells. The envelope spike glycoprotein binds to its cellular receptor, ACE2 for SARS-CoV and SARS-CoV-2, CD209L (a C-type lectin, also called L-SIGN) for SARS-CoV, DPP4 for MERS-CoV.

Virus genome sequencing of patients with pneumonia hospitalized in the month of December, 2019, disclosed the presence of a formerly unknown β-CoV strain in all of them [42]. This isolated novel β-CoV shows 88% identity to the sequence of two bat-derived severe acute respiratory syndromes (SARS)-like coronaviruses, bat-SL-CoVZC45 and bat-SL-CoVZXC21, and about 50% identity to the sequence of MERS-CoV [42]. The novel β-CoV was named “SARS-CoV-2” by the International Virus Classification Commission.

Patients with COVID-19 show clinical symptoms including fever, nonproductive cough, dyspnea, myalgia, fatigue, normal or decreased leukocyte counts, and radiographic evidence of pneumonia. One of the important mechanisms for ARDS is the cytokine storm, the deadly uncontrolled systemic inflammatory response ensuing from the release of large amounts of pro-inflammatory cytokines (IFN-a, IFN-g, IL-1b, IL-6, IL-12, IL-18, IL-33, TNF-a, TGF-b, etc.) and chemokines (CCL2, CCL3, CCL5, CXCL8, CXCL9, CXCL10, etc.).

Rapid and accurate detection of Covid-19 is crucial in controlling the outbreak within the community and in hospitals. Nasopharyngeal and oropharyngeal swabs are the suggested upper respiratory tract specimen types for Covid-19 diagnosis. The collection of these specimen types requires close contact between healthcare workers and patients, which jeopardize a risk of transmission of the virus to the healthcare workers. Furthermore, the collection of nasopharyngeal or oropharyngeal specimens causes discomfort and may cause bleeding, especially in patients with condition such as thrombocytopenia [43].

Some virus strains have been detected in saliva as long as 29 days after infection, indicating that a non-invasive platform to rapidly differentiate the biomarkers using saliva may enhance disease detection [44]. The sputum of a lower respiratory tract was produced by only 28% of COVID-19 patients, which indicates a strong limitation as specimen for diagnostic evaluation [45]. It has been previously demonstrated that saliva has a high consistency rate of greater than 90% with nasopharyngeal specimens in the detection of respiratory viruses, including coronaviruses [46].

In Hong Kong, Covid-19 testing was performed by Public Health Laboratory Services Branch for patients who fulfilled the enhanced surveillance criteria [47]. A patient is considered to have confirmed infection if Covid-19 was detected in their nasopharyngeal or sputum specimens. Saliva was collected and subjected to nucleic acid extraction and real-time reverse transcription-quantitative polymerase chain reaction for Covid-19. RT-qPCR is the gold standard for detecting pathogenic viruses in respiratory secretions and blood. A total of 12 patients with laboratory-confirmed Covid-19 infection in Hong Kong were included. Saliva specimens were collected at a median of two days after hospitalization (range, 0-7 days). Covid-19 was detected in the initial saliva specimens of 11 patients (91.7%). In 33 patients whose nasopharyngeal specimens tested negative for Covid-19, all the saliva specimens also tested negative.

Recently, researchers from RUCDR Infinite Biologics at Rutgers University have successfully validated saliva as being a viable biosample source for COVID-19 detection when compared to nasopharyngeal or oropharyngeal swabs. According to them, the utilisation of saliva to extract viral RNA was in fact a robust source for COVID-19 detection and equals in performance to the approved swab-based collection samples. Saliva testing will help with the global shortage of swabs for sampling and increase testing of patients, and it will eliminate the requirement of health care professionals to collect samples [48].

The use of saliva will permit specimen collection outside the hospitals where airborne-infection isolation rooms are not available, such as in outpatient clinics or in the community. SARS-CoV has been shown to infect epithelial cells in salivary gland ducts in rhesus macaques. The presence of Covid-19 in patients’ saliva suggests the likelihood of salivary gland infection. However, it should be noted that saliva specimens not only contain saliva secreted from major or minor salivary glands but also contain secretions coming down from the nasopharynx or coming up from the lung via the action of cilia lining the airway. More studies are needed to delineate the sources of Covid-19 in saliva.

In the setting where huge number of individuals require screening, saliva would constitute a practical noninvasive specimen type. Since healthcare workers are not required to collect saliva specimens, the use of saliva specimens will eliminate the waiting time, and therefore the results would be available much sooner. This is vital in busy clinical settings. Covid-19 may be transmitted via saliva directly or indirectly even among patients without coughing or other respiratory symptoms. Saliva specimens have high sensitivity and specificity in the detection of respiratory viruses by an automated multiplex Clinical Laboratory Improvement Amendments-waived point-of-care molecular assay when compared with those of nasopharyngeal aspirate. The use of saliva also reduces the time and cost associated with the collection of specimen [47]. Further studies are needed to evaluate the potential diagnostic of Covid-19 in saliva and its impact on transmission of this virus, which is pivotal to develop rapid diagnostic tests and effective strategies for prevention.

## Conclusions

Saliva offers many benefits as a diagnostic fluid as it is easy to collect, store and contains extremely good quality DNA. Thus, saliva can be an ideal alternative for blood. The research in the field of salivaomics has a key role in identifying biomarkers and exploring the role of saliva in diagnosis of diseases. It is anticipated that the development of precise salivary diagnostic tools will make salivary diagnostics a reality in the future. Saliva can be provided by patients without any invasive procedures, the use of saliva specimens will decrease the risk of nosocomial transmission of Covid-19 and is ideal for situations in which oro- or nasopharyngeal specimen collection may be contraindicated. It can also reduce or eliminate the need for health care professionals for collecting samples. Recent research suggests that saliva can be used as a viable biosample for the detection of Covid-19, further studies are required to validate the same. Salivary diagnostics may play a pivotal role in detection of Covid-19 and can offer mass screening of the population.

## References

[1] Zhang CZ, Cheng XQ, Li JY, Zhang P, Yi P, Xu X, Zhou XD (2016). Saliva in the diagnosis of diseases. Int J Oral Sci.

[2] Lawrence HP (2002). Salivary markers of systemic disease: noninvasive diagnosis of disease and monitoring of general health. J Can Dental Assoc.

[3] Javaid MA, Ahmed AS, Durand R, Tran SD (2016). Saliva as a diagnostic tool for oral and systemic diseases. J Oral Biol Craniofac Res.

[4] Lee YH, Wong DT (2009). Saliva: an emerging biofluid for early detection of diseases. Am J Dent.

[5] Tzioufas AG, Kapsogeorgou EK (2015). Saliva proteomics is a promising tool to study Sjögren syndrome. Nat Rev Rheumatol.

[6] Krishnamurthy S, Vasudeva SB, Vijayasarathy S (2015). Salivary gland disorders: a comprehensive review. World J Stomatol.

[7] Pfaffe T, Cooper-White J, Beyerlein P, Kostner K, Punyadeera C (2011). Diagnostic potential of saliva: current state and future applications. Clin Chem.

[8] Sabino-Silva R, Jardim AC, Siqueira WL (2020). Coronavirus COVID-19 impacts to dentistry and potential salivary diagnosis. Clin Oral Investig.

[9] Lee JM, Garon E, Wong DT (2009). Salivary diagnostics. Orthod Craniofac Res.

[10] Mittal S, Bansal V, Garg S, Atreja G, Bansal S (2011). The diagnostic role of saliva—a review. J Clin Exp Dent.

[11] Wong DT (2012). Salivaomics. J Am Dent Assoc.

[12] Tremblay M, Loucif Y, Methot J, Brisson D, Gaudet D (2012). Salivary pH as a marker of plasma adiponectin concentrations in women. Diabetol Metab Syndr.

[13] Samaranayake L (2007). Saliva as a diagnostic fluid. Int Dent J.

[14] Nishikawara F, Katsumura S, Ando A (2006). Correlation of cariogenic bacteria and dental caries in adults. J Oral Sci.

[15] O'Brien-Simpson NM, Burgess K, Brammar GC, Darby IB, Reynolds EC (2015). Development and evaluation of a saliva-based chair-side diagnostic for the detection of Porphyromonas gingivalis. J Oral Microbiol.

[16] Prescher N, Maier K, Munjal SK, Sorsa T, Bauermeister CD, Struck F, Netuschil L (2007). Rapid quantitative chairside test for active MMP-8 in gingival crevicular fluid: first clinical data. Ann N Y Acad Sci.

[17] Sorsa T, Hernández M, Leppilahti J, Munjal S, Netuschil L, Mäntylä P (2010). Detection of gingival crevicular fluid MMP‐8 levels with different laboratory and chair‐side methods. Oral Dis.

[18] Prakasam S, Srinivasan M (2014). Evaluation of salivary biomarker profiles following non‐surgical management of chronic periodontitis. Oral Dis.

[19] Karimbux NY, Saraiya VM, Elangovan S, Allareddy V, Kinnunen T, Kornman KS, Duff GW (2012). Interleukin‐1 gene polymorphisms and chronic periodontitis in adult whites: a systematic review and meta‐analysis. J Periodontol.

[20] Song GG, Choi SJ, Ji JD, Lee YH (2013). Association between tumor necrosis factor-α promoter− 308 A/G,− 238 A/G, interleukin-6− 174 G/C and− 572 G/C polymorphisms and periodontal disease: a meta-analysis. Mol Biol Rep.

[21] Wang Y, Springer S, Mulvey CL (2015). Detection of somatic mutations and HPV in the saliva and plasma of patients with head and neck squamous cell carcinomas. Sci Transl Med.

[22] Nagler R, Bahar G, Shpitzer T, Feinmesser R (2006). Concomitant analysis of salivary tumor markers—a new diagnostic tool for oral cancer. Clin Cancer Res.

[23] Li Y, John MA, Zhou X (2004). Salivary transcriptome diagnostics for oral cancer detection. Clin Cancer Res.

[24] Streckfus C, Bigler L, Tucci M, Thigpen JT (2000). A preliminary study of CA15-3, c-erbB-2, epidermal growth factor receptor, cathepsin-D, and p53 in saliva among women with breast carcinoma. Cancer Invest.

[25] Chen DX, Schwartz PE, Li FQ (1990). Saliva and serum CA 125 assays for detecting malignant ovarian tumors. Obstet Gynecol.

[26] Wei F, Lin CC, Joon A (2014). Noninvasive saliva-based EGFR gene mutation detection in patients with lung cancer. Am J Respir Crit Care Med.

[27] Hizir MS, Balcioglu M, Rana M, Robertson NM, Yigit MV (2014). Simultaneous detection of circulating oncomiRs from body fluids for prostate cancer staging using nanographene oxide. ACS Appl Mater Interfaces.

[28] Dirix P, Nuyts S, Vander Poorten V, Delaere P, Van den Bogaert W (2007). Efficacy of the BioXtra dry mouth care system in the treatment of radiotherapy-induced xerostomia. Support Care Cancer.

[29] Malathi N, Mythili S, Vasanthi HR (2014). Salivary diagnostics: a brief review. ISRN Dent.

[30] Aitken JP, Ortiz C, Morales-Bozo I, Rojas-Alcayaga G, Baeza M, Beltran C, Escobar A (2015). α-2-macroglobulin in saliva is associated with glycemic control in patients with type 2 diabetes mellitus. Dis Markers.

[31] Novak BJ, Blake DR, Meinardi S, Rowland FS, Pontello A, Cooper DM, Galassetti PR (2007). Exhaled methyl nitrate as a noninvasive marker of hyperglycemia in type 1 diabetes. Proc Natl Acad Sci USA.

[32] Strauss SM, Wheeler AJ, Russell SL (2009). The potential use of gingival crevicular blood for measuring glucose to screen for diabetes: an examination based on characteristics of the blood collection site. J Periodontol.

[33] Kosaka T, Kokubo Y, Ono T (2014). Salivary inflammatory cytokines may be novel markers of carotid atherosclerosis in a Japanese general population: the Suita study. Atherosclerosis.

[34] Out D, Hall RJ, Granger DA, Page GG, Woods SJ (2012). Assessing salivary C-reactive protein: longitudinal associations with systemic inflammation and cardiovascular disease risk in women exposed to intimate partner violence. Brain Behav Immun.

[35] Saunders JT, Nambi V, De Lemos JA (2011). Cardiac troponin T measured by a highly sensitive assay predicts coronary heart disease, heart failure, and mortality in the Atherosclerosis Risk in Communities Study. Circulation.

[36] Mirzaii‐Dizgah I, Riahi E (2013). Salivary high‐sensitivity cardiac troponin T levels in patients with acute myocardial infarction. Oral Dis.

[37] Parisi MR, Soldini L, Vidoni G (2014). Point-of-care testing for HCV infection: recent advances and implications for alternative screening. New Microbiol.

[38] Nefzi F, Ben Salem NA, Khelif A, Feki S, Aouni M, Gautheret‐Dejean A (2015). Quantitative analysis of human herpesvirus‐6 and human cytomegalovirus in blood and saliva from patients with acute leukemia. J Med Virol.

[39] Li X, Geng M, Peng Y, Meng L, Lu S (2020). Molecular immune pathogenesis and diagnosis of COVID-19. J Pharm Anal.

[40] Su S, Wong G, Shi W (2016). Epidemiology, genetic recombination, and pathogenesis of coronaviruses. Trends Microbiol.

[41] Perlman S, Netland J (2009). Coronaviruses post-SARS: update on replication and pathogenesis. Nat Rev Microbiol.

[42] Lu R, Zhao X, Li J (2020). Genomic characterisation and epidemiology of 2019 novel coronavirus: implications for virus origins and receptor binding. Lancet.

[43] Chan JF, Yuan S, Kok KH (2020). A familial cluster of pneumonia associated with the 2019 novel coronavirus indicating person-to-person transmission: a study of a family cluster. Lancet.

[44] Barzon L, Pacenti M, Berto A (2016). Isolation of infectious Zika virus from saliva and prolonged viral RNA shedding in a traveller returning from the Dominican Republic to Italy, January 2016. Euro Surveill.

[45] To KK, Tsang OT, Yip CC (2020). Consistent detection of 2019 novel coronavirus in saliva. Clin Infect Dis.

[46] To KK, Yip CC, Lai CY (2019). Saliva as a diagnostic specimen for testing respiratory virus by a point-of-care molecular assay: a diagnostic validity study. Clin Microbiol Infect.

[47] (2020). Centre for Health Protection. Severe respiratory disease associated with a novel infectious agent—letters to doctors. https://www.chp.gov.hk/en/miniweb/letters/100063.html.

[48] (2020). Rutgers launches genetic testing service for new coronavirus. https://www.rutgers.edu/news/rutgers-launches-genetic-testing-service-new-coronavirus.

